# Supplementary material for: A Toxicological Framework for the Prioritization of Children’s Safe Product Act Data
Source: Int J Environ Res Public Health. 2016 Apr 19;13(4):431. doi: 10.3390/ijerph13040431 (PMC4847093; doi:10.3390/ijerph13040431)
Supplement: Supplementary file 1 [file ijerph-13-00431-s001.pdf]

Supplementary Materials: A Toxicological Framework for the Prioritization of Children's Safe Product Act Data

Marissa N. Smith, Joshua Grice, Alison Cullen and Elaine M. Faustman

Table S1. Total number of reports in the CSPA database from August 2012 to September 2015 by chemical. Chemicals shown in bold are included in the analyses for this paper. These chemicals represent approximately 88% of the database as a whole.

Chemicals	Number of Reports
**Cobalt & cobalt compounds**	**6927**
**Ethylene glycol**	**6042**
**Antimony & Antimony compounds**	**3378**
**Methyl ethyl ketone**	**2378**
**Styrene**	**2251**
**Octamethylcyclotetrasiloxane**	**2123**
**Molybdenum & molybdenum compounds**	**1617**
**Di-2-ethylhexyl phthalate**	**909**
**Dibutyl phthalate**	**778**
**Butyl benzyl phthalate (BBP)**	**610**
**Formaldehyde**	**533**
Toluene	495
Ethylbenzene	458
Arsenic & Arsenic compounds arsenic trioxide (1327-53-3) & dimethyl arsenic (75-60-5)	387
Diethyl phthalate	380
Diisononyl phthalate (DINP)	357
4-Nonylphenol; 4-NP and its isomer mixtures CAS 84852-15-3 and CAS 25154-52-3	338
Cadmium & cadmium compounds	282
Di-n-octyl phthalate (DnOP)	279
Methyl paraben	251
Diisodecyl phthalate (DIDP)	235
Acetaldehyde	216
Propyl paraben	207
Di-n-Hexyl phthalate	178
C.I. solvent yellow 14	172
Mercury & mercury compounds including methyl mercury (22967-92-6)	165
2-Ethylhexanoic acid	154
Phenol	140
Phthalic anhydride	137
Ethyl paraben	97
n-Butanol	95
Bisphenol A	87
Butyl paraben	83
Acrylonitrile	73
Methylene chloride	70
Tetrabromobisphenol A	62
Vinyl chloride	52
Aniline	45
Carbon disulfide	42
2,2′,3,3′,4,4′,5,5′,6,6′-Decabromodiphenyl ether; BDE-209	41
3,3'-Dimethylbenzidine and Dyes Metabolized to 3,3'-Dimethylbenzidine	39
Benzene	37
2-Ethyl-hexyl-4-methoxycinnamate	32
Ethylene glycol monoethyl ester	31
p-Hydroxybenzoic acid	31
Estragole	28
2-Methoxyethanol	26
4-tert-Octylphenol; 1,1,3,3-Tetramethyl-4-butylphenol	23
Tris(2-chloroethyl) phosphate	23
Hexachlorobutadiene	21

Table S1. *Cont.*

Chemicals	Number of Reports
N-Methylpyrrolidone	21
2,4-Diaminotoluene	20
Perfluorooctanyl sulphonic acid and its salts; PFOS	20
1,4-Dioxane	19
Phenol, 4-octyl-	19
2-Aminotoluene	18
Hexabromocyclododecane	18
N-Nitrosodimethylamine	18
N-Nitrosodiphenylamine	18
Perchloroethylene	18
1,1,2,2-Tetrachloroethane	17
para-Chloroaniline	17
Benzene, pentachloro	16
Butylated hydroxyanisole; BHA	16
Hexachlorobenzene	14
Tris(1,3-dichloro-2-propyl)phosphate	5
Benzophenone-2 (Bp-2); 2,2′,4,4′-Tetrahydroxybenzophenone	3
Total Number of Records	33,692

Table S2. Potency factor scores and sources for reproductive and developmental toxicants and endocrine disruptors.

Chemical	Reproductive and Developmental Toxicants			Endocrine Disruptors			
	NOAEL	Score	Endpoint Examined and Reference	Reported LOAEL	Calculated NOAEL	Score	Endpoint Examined and Reference
**BBP**	50 mg/kg/day	3	Anogenital distance (AGD) in both F1 and F2 *	1000 mg/kg/day	100	3	Testis impacts #
**Butyl Paraben**				100 mg/kg/day	10	3	Decreased sperm count #
**DEHP**	4.8 mg/kg/day	3	Irreversible testis damage *	52 mg/kg/day	5.2	3	Induction of peroxisome proliferation #
**DBP**	52 mg/kg/day	3	Embryotoxicity *	50 mg/kg/day	5	3	Testis decreased weight # or increased weight #
**DEP**				2000 mg/kg/day	200	3	Leydig cell ultra-structural alterations #
**DIDP**	33 mg/kg/day	3	Decreased offspring survival *				
**DINP**	200 mg/kg/day	3	Fetal toxicity *				
**DnHP**	38 mg/kg/day	3	Based on 380 mg/kg/day LOAEL ^				
**Ethyl Paraben**				1000 mg/kg/day	1000	1	NOAEL for sex hormone secretion#
**Methyl Ethyl Ketone**	594 mg/kg/day	1	Based on 0.6 RfD with a 1000X UF Study endpoint: decreased Pup Body Weight $				

* From ECHA Existing Substances Database, $ From EPA IRIS, ^ From NTP, # From ECHA Substances of Concern Database, UF is for Uncertainty Factor.

Table S3. Total priority indices, exposure scores and number of reports by product segment (gray shaded rows) are broken down by chemical groups. The three chemicals with the highest average priority index are shaded in light red. In every case except beauty/personal care/hygiene, formaldehyde, phthalates and styrene had the highest average total priority indices. Methyl ethyl ketone, formaldehyde and styrene had the highest average total priority indices for beauty/personal/care/hygiene products.

	Average Priority Index	Average Exposure Score	Number of Reports
Arts/Crafts/Needlework	105.3	9.3	631
Antimony & Antimony compounds	27.0	9.0	93
Cobalt & cobalt compounds	70.0	7.0	25
Ethylene glycol	64.0	8.0	64
Formaldehyde	241.5	11.5	28
Methyl ethyl ketone	76.5	8.5	26
Molybdenum & molybdenum compounds	0.0	5.0	27
Octamethylcyclotetrasiloxane	13.0	13.0	12
Parabens	12.0	10.0	80
Phthalates	168.3	9.4	211
Styrene	195.5	11.5	65
Baby Care	103.8	10.7	991
Antimony & Antimony compounds	31.4	10.5	110
Cobalt & cobalt compounds	91.3	9.1	174
Ethylene glycol	85.3	10.7	170
Formaldehyde	294.0	14.0	18
Methyl ethyl ketone	100.0	11.1	55
Molybdenum & molybdenum compounds	0.0	6.0	55
Octamethylcyclotetrasiloxane	13.8	13.8	45
Parabens	12.4	11.5	29
Phthalates	158.4	11.3	263
Styrene	216.3	12.7	72
Beauty/Personal Care/Hygiene	42.4	10.2	559
Antimony & Antimony compounds	28.8	9.6	36
Cobalt & cobalt compounds	70.0	7.0	30
Ethylene glycol	68.2	8.5	43
Formaldehyde	243.9	11.6	22
Methyl ethyl ketone	76.5	8.5	12
Octamethylcyclotetrasiloxane	13.0	13.0	12
Parabens	17.5	10.9	359
Phthalates	73.0	7.4	32
Styrene	198.1	11.7	13
Camping	71.1	8.9	87
Antimony & Antimony compounds	30.6	10.2	5
Cobalt & cobalt compounds	82.5	8.3	32
Ethylene glycol	76.8	9.6	25
Methyl ethyl ketone	76.5	8.5	7
Molybdenum & molybdenum compounds	0.0	5.4	10
Octamethylcyclotetrasiloxane	13.0	13.0	4
Styrene	221.0	13.0	4
Clothing	79.2	9.3	14,551
Antimony & Antimony compounds	29.2	9.7	1703
Cobalt & cobalt compounds	81.3	8.1	4329
Ethylene glycol	75.1	9.4	3691
Formaldehyde	264.8	12.6	185
Methyl ethyl ketone	87.0	9.7	928
Molybdenum & molybdenum compounds	0.0	5.6	879
Octamethylcyclotetrasiloxane	13.5	13.5	805
Parabens	35.7	11.6	57
Phthalates	134.1	10.0	1092
Styrene	209.1	12.3	882
Footwear	90.5	10.0	4940
Antimony & Antimony compounds	29.2	9.7	322
Cobalt & cobalt compounds	80.9	8.1	922
Ethylene glycol	74.8	9.3	804
Formaldehyde	253.2	12.1	18

Table S3. *Cont.*

	Average Priority Index	Average Exposure Score	Number of Reports
Methyl ethyl ketone	88.7	9.9	785
Molybdenum & molybdenum compounds	0.0	5.5	190
Octamethylcyclotetrasiloxane	13.6	13.6	753
Parabens	33.0	10.5	8
Phthalates	187.6	10.2	909
Styrene	208.2	12.2	229
Household/Office Furniture/Furnishings	105.1	10.7	1446
Antimony & Antimony compounds	30.7	10.2	165
Cobalt & cobalt compounds	86.5	8.6	238
Ethylene glycol	79.6	10.0	234
Formaldehyde	269.5	12.8	73
Methyl ethyl ketone	99.0	11.0	141
Molybdenum & molybdenum compounds	0.0	5.9	64
Octamethylcyclotetrasiloxane	14.0	14.0	141
Parabens	36.0	11.8	4
Phthalates	177.0	11.3	297
Styrene	217.0	12.8	89
Kitchen Merchandise	205.8	12.2	72
Antimony & Antimony compounds	31.5	10.5	6
Cobalt & cobalt compounds	90.0	9.0	4
Formaldehyde	294.0	14.0	12
Molybdenum & molybdenum compounds	0.0	6.0	2
Parabens	0.0	11.0	1
Phthalates	232.0	12.2	32
Styrene	221.0	13.0	15
Personal Accessories	82.3	9.0	1229
Antimony & Antimony compounds	27.6	9.2	88
Cobalt & cobalt compounds	73.1	7.3	294
Ethylene glycol	67.6	8.5	236
Formaldehyde	241.5	11.5	22
Methyl ethyl ketone	80.6	9.0	137
Molybdenum & molybdenum compounds	0.0	5.1	90
Octamethylcyclotetrasiloxane	13.2	13.2	107
Phthalates	142.4	9.6	127
Styrene	198.5	11.7	128
Stationery/Office Machinery/Occasion Supplies	158.6	10.1	365
Antimony & Antimony compounds	28.0	9.3	37
Cobalt & cobalt compounds	73.8	7.4	24
Ethylene glycol	64.0	8.0	25
Formaldehyde	284.6	13.6	10
Methyl ethyl ketone	76.5	8.5	17
Molybdenum & molybdenum compounds	0.0	5.0	10
Octamethylcyclotetrasiloxane	13.0	13.0	12
Parabens	0.0	10.2	5
Phthalates	220.5	10.6	199
Styrene	203.7	12.0	26
Toys/Games	131.9	13.0	4910
Antimony & Antimony compounds	36.5	12.2	813
Cobalt & cobalt compounds	108.0	10.8	855
Ethylene glycol	102.1	12.8	781
Formaldehyde	389.1	18.5	145
Methyl ethyl ketone	118.5	13.2	270
Molybdenum & molybdenum compounds	0.0	6.9	290
Octamethylcyclotetrasiloxane	16.6	16.6	232
Parabens	25.4	13.1	95
Phthalates	210.9	13.4	701
Styrene	279.0	16.4	728

© 2016 by the authors; licensee MDPI, Basel, Switzerland. This article is an open access article distributed under the terms and conditions of the Creative Commons by Attribution (CC-BY) license (http://creativecommons.org/licenses/by/4.0/).
